# The Effect of Fructose Feeding on Intestinal Triacylglycerol Production and De Novo Fatty Acid Synthesis in Humans

**DOI:** 10.3390/nu12061781

**Published:** 2020-06-15

**Authors:** Simon Steenson, Fariba Shojaee-Moradie, Martin B. Whyte, Kim G. Jackson, Julie A. Lovegrove, Barbara A. Fielding, A. Margot Umpleby

**Affiliations:** 1 Department of Nutritional Sciences, Faculty of Health and Medical Sciences, University of Surrey, Guildford GU2 7WG, UK; simon.j.steenson@gmail.com (S.S.); f.shojaee-moradie@surrey.ac.uk (F.S.-M.); m.b.whyte@surrey.ac.uk (M.B.W.); b.fielding@surrey.ac.uk (B.A.F.); 2 Food & Nutritional Sciences and Institute for Cardiovascular and Metabolic Research (ICMR), University of Reading, Reading RG6 6AP, UK; k.g.jackson@reading.ac.uk (K.G.J.); j.a.lovegrove@reading.ac.uk (J.A.L.)

**Keywords:** chylomicrons, VLDL, kinetics, stable isotope tracers, apolipoprotein B100, apolipoprotein B48, intestinal de novo lipogenesis, hepatic de novo lipogenesis, postprandial

## Abstract

A high fructose intake exacerbates postprandial plasma triacylglycerol (TAG) concentration, an independent risk factor for cardiovascular disease, although it is unclear whether this is due to increased production or impaired clearance of triacylglycerol (TAG)-rich lipoproteins. We determined the in vivo acute effect of fructose on postprandial intestinal and hepatic lipoprotein TAG kinetics and de novo lipogenesis (DNL). Five overweight men were studied twice, 4 weeks apart. They consumed hourly mixed-nutrient drinks that were high-fructose (30% energy) or low-fructose (<2% energy) for 11 h. Oral ^2^H_2_O was administered to measure fasting and postprandial DNL. Postprandial chylomicron (CM)-TAG and very low-density lipoprotein (VLDL)-TAG kinetics were measured with an intravenous bolus of [^2^H_5_]-glycerol. CM and VLDL were separated by their apolipoprotein B content using antibodies. Plasma TAG (*p* < 0.005) and VLDL-TAG (*p* = 0.003) were greater, and CM-TAG production rate (PR, *p* = 0.046) and CM-TAG fractional catabolic rate (FCR, *p* = 0.073) lower when high-fructose was consumed, with no differences in VLDL-TAG kinetics. Insulin was lower (*p* = 0.005) and apoB48 (*p* = 0.039), apoB100 (*p* = 0.013) and non-esterified fatty acids (NEFA) (*p* = 0.013) were higher after high-fructose. Postprandial hepatic fractional DNL was higher than intestinal fractional DNL with high-fructose (*p* = 0.043) and low-fructose (*p* = 0.043). Fructose consumption had no effect on the rate of intestinal or hepatic DNL. We provide the first measurement of the rate of intestinal DNL in humans. Lower CM-TAG PR and CM-TAG FCR with high-fructose consumption suggests lower clearance of CM, rather than elevated production, may contribute to elevated plasma TAG, possibly due to lower insulin-mediated stimulation of lipoprotein lipase.

## 1. Introduction

Elevated fasting or postprandial plasma triacylglycerol (TAG) has been shown to be an independent risk factor for the development of cardiovascular disease (CVD) [1]. Raised postprandial TAG may result from (i) overproduction of very low-density lipoproteins (VLDL), which are synthesised by the liver and contain the higher molecular weight form of apolipoprotein (apo)B, apoB100; (ii) overproduction of chylomicrons (CM), synthesised in the small intestine in response to dietary fat, which contain the lower molecular weight form of apoB (apoB48); [iii] impaired clearance of CM and/or VLDL or [iv] a combination of these processes.

Diet is an important modifiable risk factor for CVD, and high consumption of the monosaccharide fructose has been shown to increase both fasting [2,3] and postprandial TAG and apoB48 levels [4,5,6] as well as fractional hepatic de novo lipogenesis (DNL) [7,8,9]. In the Syrian golden hamster, apoB48 production rate was higher following chronic fructose feeding (3 weeks) versus chow feeding [10]. In this study, primary enterocytes were isolated from the hamsters fed the two regimes and the synthesis of de novo palmitate shown to be higher in enterocytes from fructose fed hamsters. This study supports more recent findings, showing that fructose specifically stimulates the expression of genes for DNL and apolipoprotein synthesis in small intestine enterocytes of mice, both in vitro and in vivo [11]. It is not known whether isoenergetic fructose feeding can increase intestinal DNL or CM-TAG production rate (PR) in humans. We investigated this using a validated constant-feeding methodology, to establish a postprandial steady state, and a sequential three-step antibody immunoaffinity method [12,13], which separates hepatic triacylglycerol-rich lipoproteins (TRL) from intestinal TRL. VLDL- and CM-TAG kinetics were measured using an intravenous bolus of [^2^H_5_]-glycerol, and hepatic and intestinal DNL were measured following oral administration of ^2^H_2_O.

## 2. Materials and Methods 

The study protocol was approved by the University of Surrey Research Integrity and Governance Office (reference: UEC/2015/020/FHMS) and the NHS Research Ethics Committee (REC reference: 15/LO/0891; South East Coast—Surrey). The trial was registered on the ISRCTN registry (ISRCTN93148765). Prior to initiation of study procedures, written informed consent was obtained from all participants. Inclusion criteria: Caucasian men; age, 18–50 y; BMI, 25–32 kg/m^2^; <3 sessions of aerobic exercise per week; weight stable for the previous 3 months (± 2.5 kg); normal haemoglobin (13.5–17.5 g/dL); blood pressure < 160/100 mmHg. Exclusion criteria: >32 g of alcohol/day; current smoker; fasting plasma glucose > 7 mmol/L; fasting plasma TAG > 4 mmol/L; prescribed lipid-lowering medications; history of endocrinological, cardiovascular, gastrointestinal and liver diseases or renal impairment; history of eating disorders, nausea or vomiting; relevant food allergies or intolerances (e.g., fructose).

### 2.1. Experimental Design

This was an acute dietary study with a randomised crossover design, involving two postprandial metabolic study days. On each study day, participants consumed isoenergetic high-fat drinks every hour for 11 h (204 kcal per drink), which were either high (30%) or low (<2%) in fructose, in order to elicit a steady postprandial TAG response. Following the first metabolic study day, participants underwent a 4-week washout period before completing the second study visit.

Subjects were requested to avoid consuming alcohol and not to undertake any strenuous exercise for 24 h prior to each metabolic study day. Subjects attended the Centre for Endocrinology, Diabetes and Research (Cedar) at Royal Surrey County Hospital (Guildford, UK) on the afternoon before each study day for a non-fasted blood sample, in order to determine their background level of plasma ^2^H_2_O and background enrichment of VLDL-TAG palmitate. At this visit, participants were given two bottles containing loading doses (total of 3 g/kg of body water) of ^2^H_2_O (99.8%, Cambridge Isotope Laboratories Inc., Tewksbury, MA, USA), to be consumed at 7 pm and 10 pm on the evening prior to the study day. Subjects were also provided with a low-fat standardised meal (Big Soup, 500 g, Heinz) to consume between 6 pm and 7 pm, as well as additional ad libitum drinking water (Nestlé Waters UK Ltd., Buxton, London, UK) containing 4.5 g/L ^2^H_2_O, to consume following the first ^2^H_2_O loading dose. Subjects were requested to consume no food or drinks other than those provided. 

**Figure 1:**
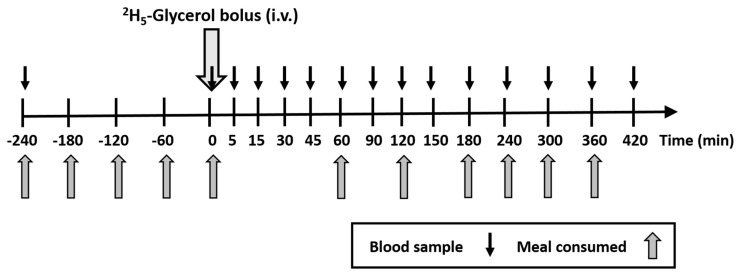
Summary of the protocol for metabolic study days.

Metabolic study protocol (Figure 1): subjects were studied the morning after a 12 h fast. A cannula was inserted into the antecubital vein of the forearm for blood sampling and a fasted blood sample was taken (−240 min). Subjects then consumed either high- or low-fructose drinks every hour for the following 11 h. Each test drink contained refined olive oil (13.4 mL; Napolina UK Ltd.), whey protein powder (8.1 g; Myprotein.com, UK) and either fructose powder (15.0 g; Tate & Lyle, UK) for the high-fructose drink or maltodextrin powder (16.0 g; Myprotein.com, UK) as a complex carbohydrate control for the low-fructose drink. Any added water contained ^2^H_2_O (5 g/L) and no added sugar orange squash concentrate (Tesco Ltd., Welwyn Garden City, UK) as a flavouring. All test drinks were made in batches <12 h before each study day and stored in sealed containers at 4 °C until required. At 0 min, 4 h after the initiation of feeding, a postprandial steady state had been established and an intravenous bolus (75 μmol/kg) of [^2^H_5_]-glycerol (30 mg/mL, 2.5 mL per vial, Pharmacy of Guy’s and St Thomas’ NHS Foundation Trust, UK) was administered. Blood samples were taken at regular intervals (Figure 1) for the isolation of TRL-TAG, the measurement of plasma glycerol enrichment, plasma ^2^H_2_O, as well as glucose, plasma TAG, non-esterified fatty acids (NEFA) and serum insulin concentrations (at hourly intervals). Blood samples were taken more frequently between 0 min and 180 min (at 5, 15, 30, 45, 60, 90, 120, 150 and 180 min) following administration of the [^2^H_5_]-glycerol bolus to allow plasma glycerol enrichment to be determined, thus allowing kinetic modelling (see data analysis section) of the incorporation of the glycerol tracer into TRL-TAG, CM-TAG and VLDL-TAG. As described previously by Shojaee-Moradie et al. [12], glycerol enrichment in lipoprotein TAG increases with time, peaking between 90 min and 120 min, and then decreases with time, and thus less frequent sampling is required. 

A fasted blood sample was taken (−240 min) before subjects consumed 11 identical high-fat (55% energy) drinks, either high- (30% energy) or low-fructose (0% energy), every hour, for 11 h. Blood samples were drawn at the times indicated with black arrows. At 0 min, an intravenous (i.v) bolus of [^2^H_5_]-glycerol (75 µmol/kg) was administered to measure lipoprotein TAG kinetics. 

Prior to each metabolic study day, participants completed a standardised food diary for the previous 4 days, in order to assess their energy and macronutrient intake. All food diaries were analysed using the Nutrition Analysis Software *Nutritics* (version 5.025; Nutritics Ltd., Dublin, Ireland). 

### 2.2. Laboratory Analyses

TAG-rich lipoproteins were separated from plasma by flotation ultracentrifugation using an LE80-K ultracentrifuge with a type 50.4 Ti fixed-angle rotor (Beckman Coulter Inc., Pasadena, CA, USA), to obtain particles with a Svedberg flotation rate (S_f_) > 20 [14]. VLDL and CM particles were isolated from the TRL samples by a sequential immunoaffinity binding method as previously described [12,13]. Three monoclonal antibodies to apoB100 (4G3, 5E11 and BSol16, Heart Institute, University of Ottawa, Ottawa, ON, Canada), coupled separately to protein G Sepharose 4 Fast flow (Amersham, UK), were used sequentially. The bound apoB100 containing VLDL fractions from each of the three sequential affinity chromatography steps were combined. The unbound apoB48 containing CM fractions were also combined. TAG in VLDL and CM fractions were extracted, purified by thin layer chromatography and hydrolysed in the presence of 3% hydrochloric acid:methanol to yield separate layers containing glycerol from TAG (TAG-glycerol) and fatty acid methyl esters (FAME). The layer containing TAG-glycerol and also plasma samples (the latter for plasma free glycerol analysis) were deproteinied and purified by ion-exchange chromatography. Freeze-dried glycerol was derivatized with N-tert-Butyldimethylsilyl-N-methyltrifluoroacetamide (MTBSTFA) to form the tert-butyldimethylsilyl (TBDMS) glycerol derivative and enrichment was measured by gas chromatography-mass spectrometry (GCMS) (Agilent 5975) in electron ionization mode. Ions monitored were *m*/*z* 377.4 and *m*/*z* 382.4 (m + 5) [15]. The [^2^H]-enrichment of the palmitate methyl ester (PAME) generated from the hydrolysis and derivatisation of TAG was measured to determine hepatic DNL (VLDL fraction) and intestinal DNL (CM fraction). Samples were analysed with a Thermo Trace 1310 GC coupled to a Delta V Advantage isotope ratio mass spectrometer, with a GC Isolink II interface (Thermo Scientific, Hemel Hempstead, UK). Plasma samples were analysed in duplicate for ^2^H_2_O enrichment with a Gasbench II inlet system and Delta V Advantage isotope ratio mass spectrometer. Platinum catalyst rods were added to the plasma samples to generate hydrogen gas from the plasma water. Sample tubes were capped and flushed (100 mL/min) with the equilibration gas, 2% H_2_ in helium and equilibrated for 6 h at 22.5 °C. Isotopic enrichment was measured relative to laboratory standards previously calibrated against the international standards Vienna Standard Mean Ocean Water 2 (VSMO2), Standard Light Arctic Precipitation (SLAP2) and Greenland Ice Sheet Precipitation (GISP) (International Atomic Energy Agency, Vienna, Austria). A series of four QCs (quality controls) of different enrichments were run with each set of samples, all of which had a mean CV < 2%.

Concentrations of fasting and postprandial plasma non-esterified fatty acids (NEFA), plasma TAG and TRL-TAG were measured by enzymatic assay (ABX, Shefford, UK) using a Cobas MIRA (Roche, Welwyn Garden City, UK). Serum insulin concentrations were measured by radioimmunoassay (Millipore Corporation, Billerica, MA, USA). ApoB48 was measured by ELISA (AKHB48, Shibayagi Corporation, Japan) and apoB-100 by an in-house ELISA.

### 2.3. Data Analysis

All tracer enrichments were expressed as tracer/tracee ratio (TTR). Using compartmental modelling, CM-TAG and VLDL-TAG fractional catabolic rate (FCR) and production rate (PR) were analysed [16]. A single-pool model was used to describe CM-TAG and VLDL-TAG kinetics with plasma glycerol as precursor pool using the SAAM II program (SAAM Institute, Seattle, WA, USA). The model represents the kinetics of the TTR profiles, which change as labelled glycerol is removed from plasma and incorporated into the TAG fractions. Plasma glycerol kinetics were described by a sum of three exponentials representing a three-compartment model. The incorporation of glycerol into VLDL by the liver and by the intestine is subject to a time delay which was described by a five-compartment chain. 

The model assumes a steady-state of native (unlabelled) glycerol throughout the experimental period. VLDL-TAG and CM-TAG production rates were calculated as the product of VLDL-TAG fractional catabolic rate (FCR) and CM-TAG FCR and their respective TAG pools. VLDL and CM-TAG pools were calculated from VLDL and CM-TAG concentration and plasma volume, as previously described [17]. 

The percent contribution of hepatic DNL to VLDL-TAG PR and intestinal DNL to CM-TAG PR (fractional DNL) was calculated from the deuterium enrichment in the palmitate of VLDL and CM-TAG, respectively, and in plasma water, as previously described [18]. The absolute contribution of DNL (mg/d) to postprandial VLDL-TAG PR and CM-TAG PR was estimated as:

*DNL contribution to VLDL-TAG PR (g/d) = % hepatic DNL × VLDL-TAG PR (g/d) × 100.*

*DNL contribution to CM-TAG PR (g/d) = % intestinal DNL × CM-TAG PR (g/d) × 100.*

### 2.4. Statistical Analysis

All time course variables were analysed using a linear mixed model to compare mean steady-state postprandial values (0–420 min) from high- and low-fructose visits. The model included estimation of the main effects for visit (visit 1 or visit 2), treatment (high- or low-fructose drink) and time (0 to 420 min), with visit-by-treatment and time-by-treatment interactions. Baseline fasting values (−240 min, fasted value) were added as a covariate. All variables were checked visually for normality of distribution using Q-Q plots and any non-normally distributed variables were log-transformed prior to analysis. One-way repeated measures ANOVA analysis was also performed for plasma TAG, CM-TAG and VLDL-TAG values for steady-state time points (0–420 min), to confirm the presence of a constant postprandial TAG concentration, necessary for kinetic calculations. Differences between fasted and mean postprandial values for high- and low-fructose visits were analysed by Wilcoxon signed-rank test. For fasted and postprandial fractional DNL data, correlations between intestinal and hepatic DNL values were compared by Spearman’s correlation analysis. All statistical analyses were performed using SPSS version 24 (IBM Corp, Armonk, NY, USA). For all tests, *p* < 0.05 was considered statistically significant. Data are mean ± SEM.

## 3. Results

### 3.1. Participant Characteristics 

Five subjects were recruited in accordance with the inclusion and exclusion criteria (mean ± SEM age 32 ± 3 y; body weight 93.1 ± 3.2 kg; BMI 27.3 ± 0.9 kg/m^2^; plasma glucose 4.8 ± 0.3 mmol/L; plasma TAG 1.28 ± 0.40 mmol/L).

Analysis of the diet diaries showed there were no significant differences in the subjects’ diet in the four days preceding the high- and low-fructose visits for any of the macronutrients analysed, including total dietary sugars, sucrose, glucose and fructose (data not shown). Mean fasting (−240 min) values were not significantly different between high-fructose and low-fructose study visits for plasma TAG or other metabolic measurements (Table 1). 

### 3.2. Postprandial Lipoprotein Kinetics

**Figure 2:**
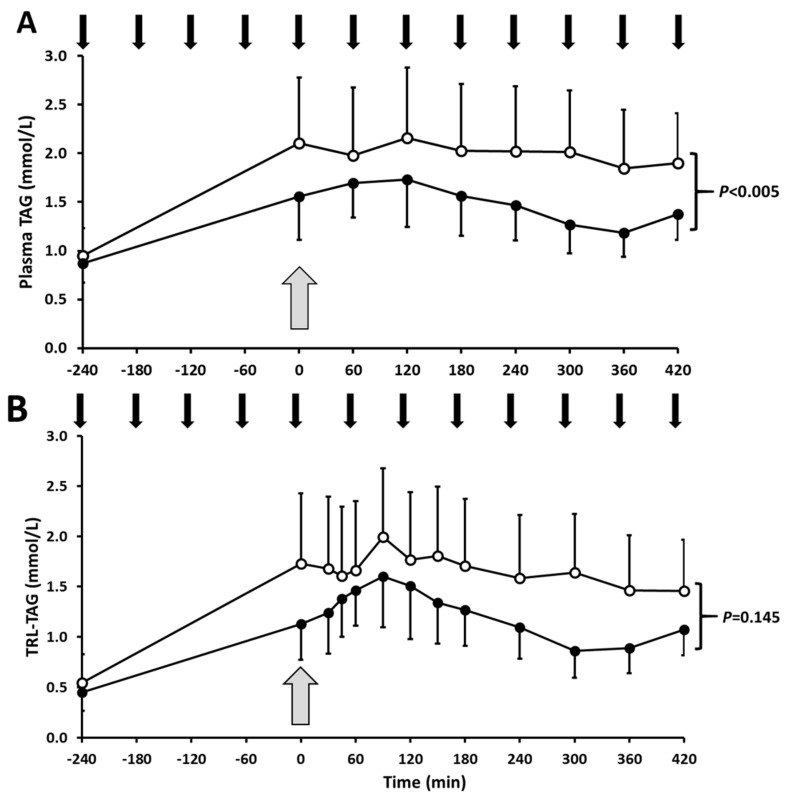
Mean plasma TAG (**A**) and TRL-TAG (**B**) responses to high- or low-fructose drinks. Subjects consumed high-fructose (white circles) or low-fructose (black circles) drinks every hour (black arrows) from fasted (−240 min) to achieve a steady postprandial state (0 min; grey arrow), at which time a bolus of [^2^H_5_]-glycerol was administered to measure lipoprotein TAG kinetics (0–420 min). Data are mean ± SEM. Time course responses were analysed by a linear mixed model to determine the treatment effect: for plasma TAG *p* < 0.005; for TRL-TAG *p* = 0.145 (NS). TAG, triacylglycerol; TRL, triacylglycerol-rich lipoproteins.

**Figure 3:**
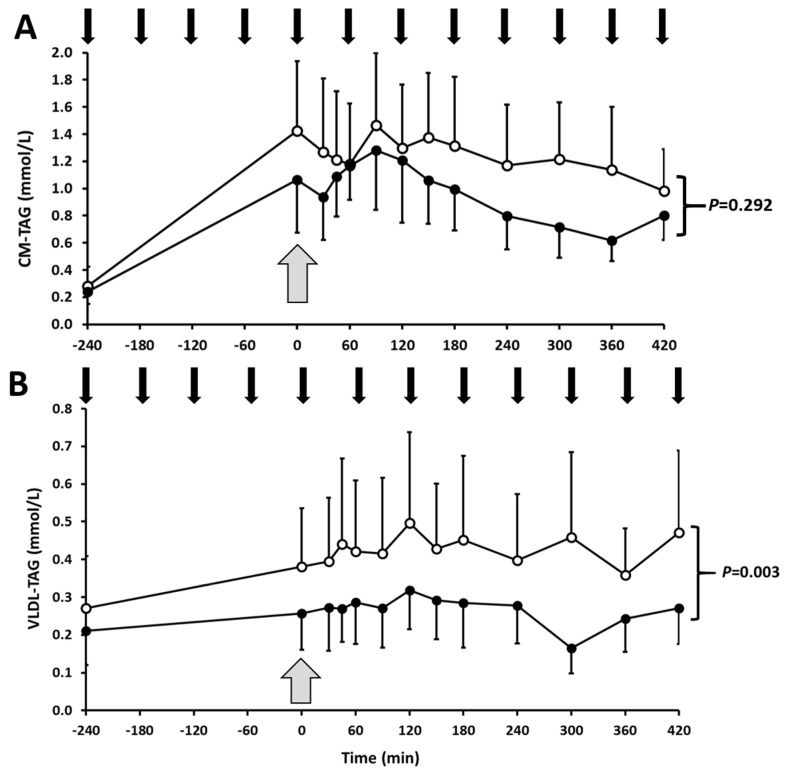
CM-TAG (**A**) and VLDL-TAG (**B**) responses to high- or low-fructose drinks. Subjects consumed high-fructose (white circles) or low-fructose (black circles) drinks every hour (black arrows) from fasted (−240 min) to achieve a steady postprandial state (0 min; grey arrow), at which time a bolus of [^2^H_5_]-glycerol was administered to measure lipoprotein TAG kinetics (0–420 min). Data are mean ± SEM (*n* = 5). Time course responses were analysed by a linear mixed model to determine the treatment effect: for CM-TAG; *p* = 0.292 (NS); for VLDL-TAG *p* = 0.003. CM, chylomicron; TAG, triacylglycerol; VLDL, very low-density lipoprotein.

Postprandial plasma TAG (Figure 2; *p* < 0.005) and VLDL-TAG (Figure 3; *p* = 0.003) concentrations were higher in response to high-fructose feeding, compared to low-fructose feeding, although no significant effect of fructose was detected for TRL-TAG (Figure 2; *p* = 0.145) or CM-TAG concentrations (Figure 3; *p* = 0.292). Mean postprandial total apoB (*p* = 0.029) (data not shown), apoB48 (*p* = 0.039) and apoB100 (*p* = 0.013) (Table 1) were all significantly higher during high-fructose feeding.

Lipoprotein TAG kinetic analysis revealed a significantly lower mean CM-TAG production rate (PR; *p* = 0.046) in response to high- versus low-fructose drinks (Table 2), which was accompanied by a lower CM-TAG FCR, although this difference did not quite attain statistical significance (*p* = 0.07). Despite the higher postprandial VLDL-TAG concentration, there were no significant differences in mean VLDL-TAG PR or FCR between study visits.

### 3.3. De Novo Lipogenesis

Fasted fractional intestinal DNL ranged from 0.40 to 2.67% across both high-fructose and low-fructose study visits, values which were quantitatively similar to those for hepatic DNL (range 0.30 to 4.17%). Postprandial fractional intestinal DNL showed little change from fasting, whereas hepatic DNL rose with feeding (an effect that was more marked with high fructose feeding, although not statistically significant). In the postprandial state, hepatic fractional DNL was significantly higher than intestinal fractional DNL, during both high and low fructose feeding (both *p* = 0.043). This difference was lost when expressed as the rate of DNL (mg/d) (Table 2).

It was noted that the within subject values for intestinal and hepatic fractional DNL were quantitatively similar during both high- and low-fructose visits. Due to the lack of any significant effect of fructose feeding, data were pooled (*n* = 10) and analysed by Spearman’s correlation analysis (data not shown), which revealed a highly significant within-subject correlation between intestinal and hepatic fractional DNL values in both the fasted (ρ = 0.939, *p* < 0.0001) and postprandial states (ρ = 0.818, *p* = 0.004), indicating a strong association between these two parameters.

### 3.4. Other Postprandial Measurements

Mean postprandial plasma glucose (*p* < 0.005) and serum insulin (*p* = 0.005) concentrations were significantly lower, and plasma NEFA remained significantly higher (*p* = 0.013), when subjects consumed high-fructose drinks, compared to low-fructose drinks. 

## 4. Discussion

Plasma TAG was higher and CM-TAG PR and FCR were lower with acute high-fructose feeding compared to low-fructose feeding. This suggests lower clearance of CM, rather than elevated production, contributed to the elevated plasma TAG concentration. This study also provides the first known estimate of the rate of intestinal DNL undertaken in human subjects and thus adds to the limited existing evidence demonstrating a DNL capability within enterocyte cells of the small intestine [19,20].

The increase in plasma TAG with high fructose feeding is in agreement with previous studies comparing acute feeding with fructose, versus either complex carbohydrate or glucose, in both men and women with BMIs ranging from normal to obese [6,21,22]. The higher VLDL-TAG with fructose feeding in our study has also been reported previously [22]. However, lipoprotein kinetic analysis in our study failed to explain the potential mechanisms for this greater VLDL-TAG excursion. Neither postprandial VLDL-TAG PR nor FCR were significantly different between the two feeding regimes, probably due to the small number of subjects and the large inter-individual variation for these measurements. It has been shown previously in seven healthy men that an intraduodenal co-infusion of fructose and intralipid (a fat emulsion intended for use in a pharmacy admixture programme) increased TRL apoB100 PR, with no effect on FCR, compared to co-infusion of glucose and lipid [23], but VLDL-TAG PR was not measured.

Although CM-TAG tended to be higher during high- versus low-fructose feeding, this difference did not reach statistical significance. Other studies have reported on the effect of fructose feeding on CM-TAG, measured as the S_f_ > 400 fraction, obtained via ultracentrifugation [9,19,20,22]. This fraction contains much larger lipoprotein particles than the S_f_ > 20 fraction isolated in the current study, and fails to isolate smaller CM, which reside in the S_f_ 60–400 density range during the later postprandial phase [24]. While in three of these studies, fructose feeding did not have a significant effect on S_f_ > 400 TAG concentration, Chong et al. (2007) reported a significantly higher CM-TAG after a fructose bolus, compared with a glucose bolus, during the later post-prandial phase [22]. The finding of a lower CM-TAG PR and FCR during high-fructose feeding in this study suggests the higher CM-TAG concentration was due to reduced clearance. Since mean postprandial serum insulin concentrations were lower during high fructose feeding, this may have resulted in lower insulin-mediated stimulation of lipoprotein lipase [25,26], thus causing a greater accumulation of CM and VLDL lipoprotein particles in the postprandial state. This is the first study to measure the specific effect of fructose on CM-TAG kinetics.

Plasma apoB48 concentrations were significantly higher with fructose feeding, indicating greater numbers of CM particles, including CM remnants. This finding is in agreement with Xiao et al. (2013), who reported increased TRL-apoB48 concentrations during intraduodenal infusion of fructose compared with glucose infusion [23]. This study also found apoB48 FCR and PR to be lower with intraduodenal fructose than with glucose, suggesting that higher mean postprandial plasma apoB48 concentrations in our study may also be due to lower FCR [23]. 

The use of antibodies in the current study to separate TRL lipoproteins of intestinal and hepatic origin, according to their specific apoB content, provides a precise measurement of both intestinal and hepatic DNL. A recent study also used antibodies to separate chylomicrons and VLDL, and measured intestinal postprandial fractional DNL [27]. Measurements in four subjects ranged from 1–4% intestinal DNL; values that were similar to the current study. 

To our knowledge, the only other human studies of intestinal DNL were in normal weight (21.9 kg/m^2^; 4 females, 4 males) [20] and obese (44.2 kg/m^2^; 5 females, 3 males) [19] subjects given mixed-nutrient meals. In both studies, [U-^13^C]-fructose given as part of the test meals was recovered as [^13^C]-palmitate in TAG in the S_f_ > 400 TRL fraction, which is typically assumed to represent CM lipoproteins of intestinal origin. It was claimed that this showed fructose may be utilised as a lipogenic substrate by enterocyte cells. However, the authors were unable to quantify the amount of de novo palmitate synthesised from fructose, as the stable isotope tracer enrichment in the acetyl-CoA precursor pool could not be determined. In addition, the S_f_ > 400 TRL fraction would have been contaminated with VLDL, as demonstrated by Jones et al. (2019) [27]. It is also possible in the above mentioned studies using [U-^13^C]-fructose that inclusion of male and female subjects may limit the ability to make comparisons with our study, in which only male subjects were studied. 

Although animal studies have shown higher intestinal DNL with fructose feeding compared with control feeding, we did not find this in the current study. In Syrian golden hamsters, an animal model of insulin resistance, the incorporation of radio-labelled [^3^H]-acetate into de novo fatty acids was three-fold greater when animals were fed a high-fructose diet (60% energy) for 3 weeks, relative to those fed a control diet of chow [10]. This may be the consequence of chronic feeding, rather than the acute fructose feeding used in our study, the presence of insulin resistance, or a different sensitivity to fructose (for use as a lipogenic substrate) between hamsters and humans.

A number of human studies have reported that chronic fructose consumption stimulates hepatic DNL [9,28], and that higher fructose doses induce a higher percentage hepatic DNL in a dose-dependent manner [7]. Hepatic DNL has also been shown to be higher in subjects following fructose feeding, than when consuming the same number of calories as either complex carbohydrate [8] or glucose [29]. However, the measurement of DNL in the S_f_ 60–400 fraction, without antibody separation of CM and VLDL, could have led to cross contamination with smaller CM and CM remnants, as shown by Jackson et al. (2002) [24], and may have accounted for a proportion of the de novo lipids. The failure to demonstrate an increase in hepatic DNL with acute fructose feeding in the current study may be due to the small number of subjects, the large inter-individual variation between subjects, or the need for chronic feeding to stimulate upregulation of key enzymes in the lipogenic pathway (e.g., acetyl-CoA carboxylase). It is also possible that fructose feeding without glucose may have directed part of the ingested fructose load away from glyceroneogenesis (to form glycerol-3-phopsphate that may be utilised for de novo TAG synthesis) and/or DNL, and towards formation of glucose via the gluconeogenic pathway.

Other limitations of the current study include the large doses of fructose (165 g/d), maltodextrin (176 g/d) and fat (135 g/d) administered to participants during metabolic study visits, which would typically be considered supra-physiological, and may only feasibly be consumed occasionally by a small proportion of the population, who are among the highest consumers. It should also be noted that fructose in the diet is typically co-ingested with glucose, and often in approximately equimolar amounts within sucrose (bound to glucose) or high-fructose corn syrup (as a free monosaccharide). Therefore, ingestion of a large amount of fructose in isolation is less physiologically feasible than in the presence of a proportion of carbohydrate as glucose.

Although the subjects had fasting metabolic measurements (including fasting glucose and plasma TAG) taken at the beginning of each metabolic study visit, which did not reveal any significant changes between visits, it is possible that alterations in their diet, physical activity levels and other lifestyle factors, during the four-week washout period, may have influenced their responses to the high- and low-fructose drinks. While the four-day food diaries indicated there were no differences in the subjects’ diet immediately prior to each visit, this did not necessarily capture their true habitual diet, due to potential issues of under-reporting or changing the foods consumed during this period to appear more acceptable [30]. 

Lastly, only a single ultracentrifugation step was used to isolate lipoproteins residing in the S_f_ > 20 range, meaning it was not possible to differentiate between VLDL_1_ (S_f_ 60–400) and VLDL_2_ (S_f_ 20–60) lipoprotein subclasses. Our laboratory has previously shown a difference in VLDL production in response to a high sugar diet (26% energy) in men with non-alcoholic fatty liver disease (NAFLD), compared to low liver fat control subjects [31]. While in control subjects a high sugar diet channelled hepatic TAG toward a higher production of VLDL_1_-TAG, patients with NAFLD exhibited a greater production of VLDL_2_-TAG. It is possible that additional separation of these two lipoprotein subclasses in the current study may have revealed differences in their regulation in response to a high fructose load, providing additional insight into the metabolic effects of fructose on postprandial TAG metabolism.

In summary, our findings indicate that intestinal and hepatic DNL made only a minor contribution to CM-TAG and VLDL-TAG production rate, respectively, in healthy subjects during high-dose acute fructose feeding. However, the highly significant within-subject correlations between intestinal and hepatic DNL, both in the fasted and postprandial states, suggests that intestinal DNL is upregulated in concert with hepatic DNL. If a similar relationship between rates of hepatic and intestinal DNL occurs in subjects with insulin resistance, the intestine may make a significant contribution to plasma TAG levels, as hepatic DNL is enhanced several-fold in these individuals [32,33]. Further studies are needed to confirm this.

## Figures and Tables

**Table 1:** Fasting and mean postprandial (0–420 min) plasma glucose, serum insulin, plasma NEFA, TRL apoB48 and TRL apoB100 in response to high- and low-fructose drinks. Values are mean ±SEM (*n* = 5).

	High-Fructose	Low-Fructose	*p*-Value
Fasting glucose (mmol/L)	4.63 ± 0.26	4.76 ± 0.12	0.495
Fasting insulin (pmol/L)	65.6 ± 12.4	85.4 ± 20.0	0.063
Fasting NEFA (mmol/L)	0.69 ± 0.17	0.81 ± 0.13	0.492
Fasting apoB48 (mg/L)	1.2 ± 0.5	1.1 ± 0.5	0.860
Fasting apoB100 (mg/L)	18.1 ± 5.4	19.7 ± 7.5	0.812
Fasting Hepatic % DNL	2.26 ± 0.65	1.30 ± 0.51	0.269
Fasting Intestinal % DNL	1.75 ± 0.46	1.27 ± 0.42	0.284
Postprandial glucose (mmol/L)	5.21 ± 0.09	5.67 ± 0.23	**<0.005**
Postprandial insulin (pmol/L)	113.0 ± 16.5	171.5 ± 31.5	**<0.005**
Postprandial NEFA (mmol/L)	0.38 ± 0.06	0.35 ± 0.05	**0.013**
Postprandial apoB48 (mg/L)	5.48 ± 2.11	3.35 ± 0.79	**0.039**
Postprandial apoB100 (mg/L)	23.89 ± 4.66	19.19 ± 6.26	**0.013**
Hepatic postprandial % DNL	4.36 ± 1.85	2.21± 1.36	0.308
Intestinal postprandial % DNL	2.33 ± 1.05	1.08 ± 0.24	0.100

Abbreviations: apoB48, apolipoprotein B48; apoB100, apolipoprotein B100; DNL, de novo lipogenesis; NEFA, non-esterified fatty acids; TRL, triacylglycerol-rich lipoproteins. High- and low-fructose treatments were compared using a paired-samples t-test (significant values *p* < 0.05 indicated in bold).

**Table 2:** Postprandial CM-TAG and VLDL-TAG kinetics and DNL in response to high- and low-fructose drinks. Values are mean ± SEM (*n* = 5).

	High-Fructose	Low-Fructose	*p*-Value
CM-TAG PR (mg/kg/d)	152.9 ± 76.6	375.93 ± 68.8	**0.046**
CM-TAG FCR (pools/d)	7.44 ± 4.01	21.69 ± 7.60	0.073
CM-TAG PS (mg)	2976 ± 1060	2247 ± 653	0.367
Intestinal DNL (mg/d)	216 ± 78	318 ± 68	0.225
VLDL-TAG PR (mg/kg/d)	81.03 ± 19.57	68.43 ± 12.18	0.641
VLDL-TAG FCR (pools/d)	10.35 ± 3.50	12.21 ± 2.78	0.254
VLDL-TAG PS (mg)	1042 ± 460	634 ± 217	0.072
Hepatic DNL (mg/d)	476 ± 310	165 ± 64	0.138

High- and low-fructose treatments were compared using a paired-samples t-test (significant values *p* < 0.05 indicated in bold). Abbreviations: CM, chylomicron; DNL, de novo lipogenesis; FCR, fractional catabolic rate; PR, production rate; PS, pool size.
